# Exploring Carbonate Rock Dissolution Dynamics and the Influence of Rock Mineralogy in CO_2_ Injection

**DOI:** 10.1021/acs.est.3c06758

**Published:** 2024-01-17

**Authors:** Javad Shokri, Matthias Ruf, Dongwon Lee, Saleh Mohammadrezaei, Holger Steeb, Vahid Niasar

**Affiliations:** † Department of Chemical Engineering, University of Manchester, Oxford Road, Manchester M13 9PL, U.K.; ‡ Institute of Applied Mechanics (MIB), Pfaffenwaldring 7, Stuttgart 70569, Germany

**Keywords:** reaction upscaling, carbon storage, fractured carbonate reservoir, dissolution-altered layer, micro-CT experiment

## Abstract

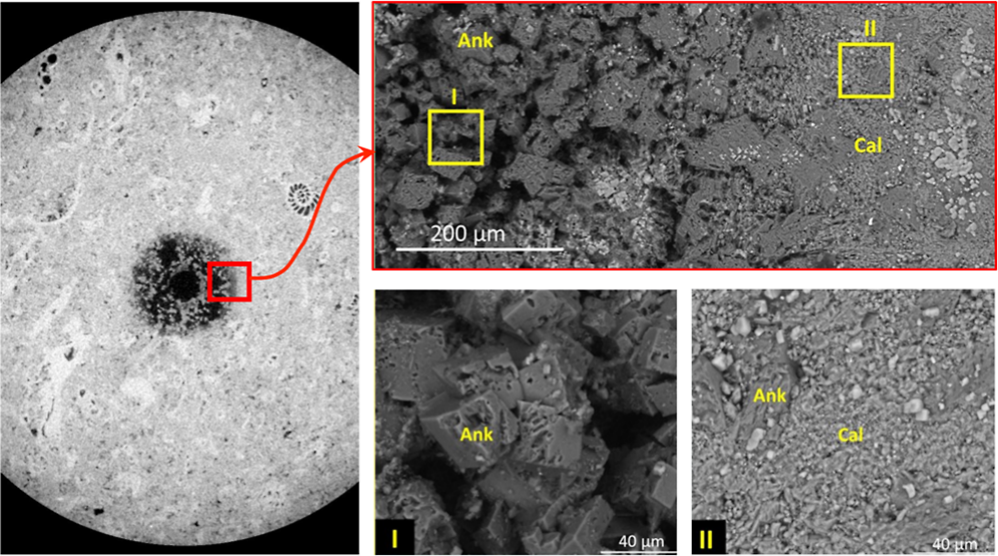

Understanding geochemical dissolution in porous materials is crucial, especially in applications such as geological CO_2_ storage. Accurate estimation of reaction rates enhances predictive modeling in geochemical-flow simulations. Fractured porous media, with distinct transport time scales in fractures and the matrix, raise questions about fracture-matrix interface dissolution rates compared to bulk dissolution rate and the scale-dependency of reaction rate averaging. Our investigation delves into these factors, studying the impact of flow rate and mineralogy on interface dissolution patterns. By injecting carbonated water into carbonate rock samples containing a central channel (mimicking fracture hydrodynamics), our study utilized μCT X-ray imaging at 3.3 μm spatial resolution to estimate the reaction rate and capture the change in pore morphology. Results revealed dissolution rates significantly lower (up to 4 orders of magnitude) than batch experiments. Flow rate notably influenced fracture profiles, causing uneven enlargement at low rates and uniform widening at higher ones. Ankerite presence led to a dissolution-altered layer on the fracture surface, showing high permeability and porosity without greatly affecting the dissolution rate, unlike clay-rich carbonates. This research sheds light on controlling factors influencing dissolution in subsurface environments, critical for accurate modeling in diverse applications.

## Abstract 2

This study offers insights into the scale-dependent nature of geochemical reaction rates and the role of rock mineralogy in geological carbon storage processes, with implications for mitigating global warming.

## Introduction

CO_2_ sequestration in geological systems, particularly saline aquifers, stands as a widely acknowledged and implemented technology aimed at curbing carbon emissions into the atmosphere.^1^ The injection of CO_2_ into saline aquifers leads to its miscibility with brine, causing a reduction in water pH. This pH decrease may trigger further dissolution of rocks, altering porosity and permeability. Hence, estimating dissolution rates becomes crucial not only for predictive modeling but also for assessing the integrity of the caprock-reservoir due to ensuing geochemical reactions.^2^ Previous studies have highlighted the scale-dependent nature of dissolution, precipitation, and reaction rates within porous media. Notably, reported geochemical reaction rates at large or field scales were considerably smaller than the rates observed in laboratory batch experiments. For instance, disparities were observed in mineral dissolution during hydrochloric acid injection^3^ and CO_2_-induced mineral dissolution^4,5^ and geothermal-induced reactions.^6^ This variance primarily stems from differences in reactant and material abundance as well as the absence of flow pathways in batch experiments. In porous media flow, the tortuosity of the media and spatial transport limitations result in higher reaction rates that are smaller than those observed in batch experiments. A comprehensive exploration of the significance of scale-dependent reaction rates in reactive transport within geological media can be found in the review paper.^7^

Upscaling the reactions in porous media becomes even more important in fractured or heterogeneous systems due to the discrepancies in the time scales of transport in high and low permeability zones. A notable example is the naturally fractured carbonate reservoirs, which are widespread globally in saline aquifers.^8−10^ While in the fractures, the transport of reactant is fast, diffusion of the reactant into the matrix is slow. This poses the question of how the fracture geometry and matrix interface would be altered by the advection and reaction time scales and whether the dissolution rate of the fracture-matrix interface is significantly different from the bulk dissolution rate.

Dissolution or reactions are controlled by the interplay between the time scale of flow, transport, and reactions, characterized by two dimensionless numbers, the Péclet (*Pe*) and Damköhler (*Da*) numbers. The Péclet number demonstrates the ratio of advective to diffusive transport, and the Damköhler number delineates the ratio of reaction rate to the transport rate. In situations with low *Pe* and high *Da* numbers, where the seepage velocity is low but the reaction time scale is much shorter than the transport time scale, the fluid reacts with minerals upon entering the fracture. This results in a face dissolution pattern and leads to a heterogeneous aperture widening along the fracture length. Increasing the *Pe* number transitions the pattern to channel formation, where most enlargements occur in areas with a higher fluid velocity. Conversely, reducing the *Da* number leads to homogeneous dissolution across the width and length of the fracture.^11−16^ The expansion of fracture aperture results in increased permeability.^17−20^ However, significant permeability reduction can occur when the fracture becomes partially or completely blocked due to particle decohesion and mobilization,^21^ or when the fracture walls close under the influence of overburden stress.^22^

Although the impact of physical factors and flow dynamics has been extensively studied in previous studies, the impact of rock mineralogy on the upscaled dissolution rate and the change of fracture morphology due to the dissolution at different dynamic conditions has been less studied. The mineralogy of the rock matrix is a critical factor as the heterogeneity of the dissolution of minerals added another level of complexity to the system, which may lead to change in dissolution patterns. For example, calcite tends to dissolve preferentially at a faster rate compared to minerals such as quartz, dolomite, and clay minerals, which leads to variations in the reaction rate in rocks containing multiple minerals.^22−25^ The presence of substantial clay content in rocks can contribute to the formation of dissolution-altered layers on fracture walls, which further complicates the estimation of upscaled dissolution rates and permeability.^26^ These layers restrict diffusion and advection, resulting in lower reaction rates.^21,27^ However, it has not been reported whether the dissolution-altered layer is exclusively found in clay-bearing carbonate rocks or if other mineral compositions could also result in its formation. Additionally, our objective is to demonstrate, for the first time as far as our knowledge extends, the upscaled reaction rate through experimental estimation. This study, employing X-ray imaging of flow experiments conducted on two distinct types of carbonate rocks. We endeavor to explore the following research objectives.

- Impact of rock mineralogy on the dissolution of fractures and the creation of a dissolution-altered layer during the injection of CO_2_-saturated water (carbonated water—CW).
- Discrepancy between the effective dissolution rate in a fractured rock compared to the bulk dissolution rates.
- Variability of the volume—averaging dissolution rates with the domain size.
- Consolidating the previously reported results about the impact of flow rate on the evolution of the fracture geometry.

## Materials and Methods

The experiments entail the injection of CW at various flow rates through cylindrical core plugs containing a centrally drilled channel. MicroCT X-ray imaging and X-ray diffraction (XRD) analysis were conducted to quantify the dissolution rate, alterations in channel morphology, and mineral composition of the rocks.

### Rock Samples

**Figure 1:**
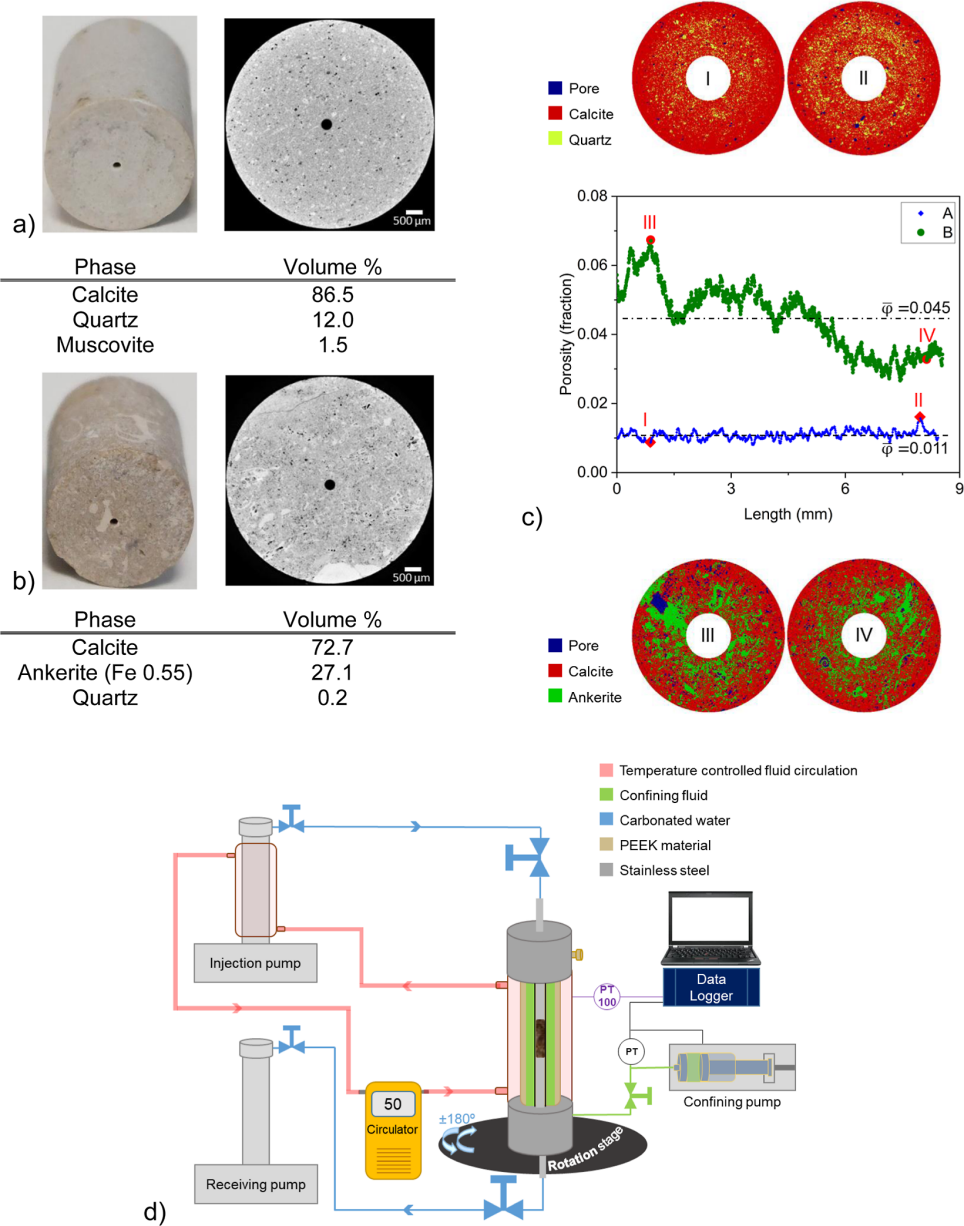
Optical images of core plugs, 2D cross-section μXRCT images along with the phase composition (volume %) of (a) sample A, and (b) sample B are presented. Both samples had a nominal diameter of 6.4 mm and a length of approximately 8.5 mm. The channel (fracture) drilled in both samples has a nominal diameter of 300 μm, (c) the porosity profile for rock types A and B are presented along with two segmented cross sections for each sample presenting mineral and pore distribution. (d) Schematic of the experimental setup is illustrated which consists of a flow cell, two ISCO pumps, a CETONI syringe pump, a heating circulator, an X-ray source (not shown), a rotation stage, and Dexela 1512 flat panel detector (not shown). The flow path of CW is represented by the blue color, while the green color represents the area filled with a confining liquid. The medium containing the heating liquid is colored pink.

Cylindrical core plugs with a diameter of 6.4 mm and length of 8.5 mm from two carbonate rock types were used in the experiments. To resemble the huge permeability contrast between the fracture and matrix, a single channel, 300 μm in diameter, was drilled at the center of the cylinders. Moreover, it enables the initialization of all core plugs with nearly identical geometries, facilitating comparisons across the experiments. XRD analysis results are shown in Figure 1a,b. These figures include gray scale cross sections obtained via μCT X-ray imaging, illustrating phase compositions of each rock type. Figure 1c displays mineral distribution in cross sections, segmented by gray scale values, alongside porosity profiles. Experimental gravimetric measurements resulted in porosity of 9% for sample A and 9.6% for sample B. The porosity profiles, coupled with mineralogy mapping, indicate a homogeneous microstructure with fine pore size in sample A, while sample B exhibits heterogeneous microstructure and mineral distribution. Detailed explanation of sample preparation is reported in the Supporting Information.

### Experimental Apparatus and Method

The experimental configuration is depicted in Figure 1c and further elaborated in the Supporting Information, which also details the experimental procedure. Table 1 outlines the experimental cases. The lower flow rate experiment spanned 5500 min, equal to 9000 IFV (injected volume of CW divided by the initial channel volume). In contrast, the higher flow rate experiments continued for 2300 min corresponding to 28 000 IFV.

**Table 1:** Details of the Experimental Cases^a^

ID	sample type	*T* (°C)	flow rate (μl min^–1^)	*Pe*	*Da*
A-25-1	A	25	1	2216	7.1 × 10^–2^
A-25-10	A	25	10	24 304	6.5 × 10^–3^
A-50-1	A	50	1	2430	9.5 × 10^–2^
A-50-10	A	50	10	23 633	9.8 × 10^–3^
B-50-1	B	50	1	2155	1.0 × 10^–1^
B-50-10	B	50	10	23 520	9.4 × 10^–3^

a *Pe* and *Da* have been reported based on the initial channel diameter. With dissolution, as the channel diameter increases with time, *Pe* and *Da* change too.

Evolution of the channel was tracked through continuous time-lapse imaging via μXRCT.^28−30^ Details of the 3D imaging and scan specifications are provided in the Supporting Information. Reconstructed images covered the full channel length and the sample width, with a 6.5 μm isotropic voxel size. Images underwent total variation filtering^31,32^ for edge-preserving denoising. Subsequently, the Otsu method,^33^ an automated threshold segmentation technique, was employed for segmenting solid and void phases that was used for quantifying channel evolution across position and time. Further discussion on the effectiveness of the imaging and the segmentation method is provided in the Supporting Information.

### Fluid Flow Simulation

The multirelaxation time lattice Boltzmann Method (MRT-LBM) was utilized to simulate single-phase fluid flow in the channel and computed permeability on segmented images. Details about the D3Q19 model can be found in previous studies.^34−36^ Solid phases were treated as bounce-back nodes, while void phases were classified as fluid nodes.^37^ A slight pressure gradient was maintained across the system by regulating the inlet and outlet pressures, and no-slip boundary conditions were applied at the solid–fluid interface. Simulations ran until reaching a steady state, followed by applying Darcy’s law to compute effective permeability for each sample based on the pressure drop and flow rate resulted from the MRT-LBM model.^38,39^

### Péclet, Damköhler Numbers, and Upscaled Dissolution Rate

Dimensionless numbers, notably the Péclet number (*Pe*) comparing advection to diffusion rates 1, and the Damköhler number (*Da*) representing reaction and advection rates 1, are crucial in interpreting governing force interactions.^14,40,41^ These numbers have been defined based on the flow and reaction in the channel, as follows
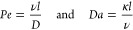
1

The kinetic rate κ [s^–1^] = *rA*_s_M_s_^14^ quantifies mineral dissolution speed, formed by the mineral’s intrinsic reaction rate *r* [mol m^–2^ s^–1^], specific surface area *A*_s_ [m^2^ kg^–1^], and the mineral molecular weight *M*_s_ [kg mol^–1^]. Additionally, ν [m s^–1^] denotes average fluid velocity in the channel, *l* [m] indicates channel length, and *D* = 7.5 × 10^–10^ m^2^ s^–1^ is the molecular diffusion for Ca^2+^.^15^*Pe* and *Da* numbers at the beginning of the experiments are presented in Table 1, with further specifics available in the Supporting Information.

Our study explores two approaches for calculating upscaled reaction rates. Conventionally, upscaling is done by averaging across entire domain of simulations^4,5,42^ or experiments^43−45^ using volume or surface averaging methods. However, we investigate the potential influence of spatial location and averaging length on these upscaled values, with further details provided in the Supporting Information.

The upscaled dissolution rate (*R* [mol m^–2^ s^–1^]) is derived from eq 2. It yields the dissolution rate over the entire channel length (denoted as *R*_1_), two rates for the half-length of the fracture (denoted as *R*_0.5_), and four rates for each quarter of the channel (denoted as *R*_0.25_). It is expressed as
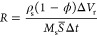
2where, ρ_*s*_ [kg m^–3^] stands for the mineral density, ϕ denotes rock matrix porosity, M_s_ [kg mol^–1^] represents molecular weight, and Δ*V*_*r*_ signifies the total volume change due to reactive mineral removal. *S̅* and Δ*t* denote the average fracture surface area and the time difference between the two consecutive scans, respectively.

## Results and Discussion

### Evolution of the Channel Geometry

**Figure 2:**
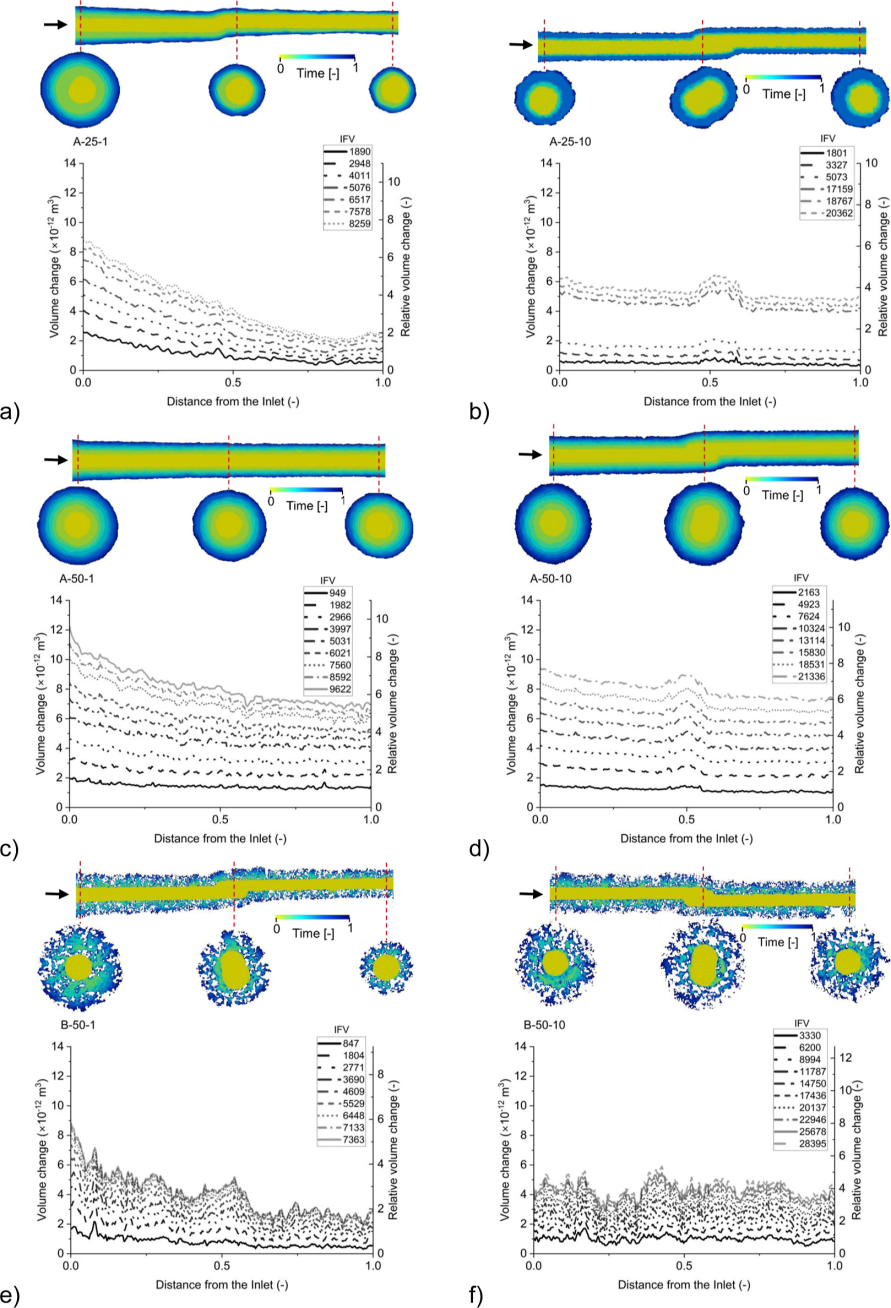
Changes of channel volume and relative volume change profiles over time for all experiments at various IFVs (Injected Volume of CW divided by the initial channel volume). They showcase channel profiles across two spatial scales—lengthwise and cross-sectional views at specific points—for each experiment, illustrating the temporal expansion of the channel. The transition from yellow to blue signifies the experiment’s temporal progression from its commencement to its conclusion. Panels a-f correspond to experiments A-25-1, A-25-10, A-50-1, A-50-10, B-50-1, and B-50-10, respectively. The label format is “sample type-temperature (°C)-flow rate (μL min^–1^)”.

Figure 2 demonstrates the temporal evolution of the channel volume, computed through image processing. Furthermore, it showcases the channel profile along the central axis in the flow direction, as well as three cross-sectional views perpendicular to the flow direction. The initial channel profile at the start of the experiment is depicted in yellow, while the final stage is represented by navy blue. Furthermore, Figure S4 shows the total cumulative channel volume change during each experiment and the relative volume change of the channel (defined as the volume change divided by the initial channel volume).

Regarding the effect of temperature, the results indicate that sample “A” exhibited greater channel volume change at higher temperatures, as anticipated based on the disparity in intrinsic reaction rates reported in previous literature.^46^ However, it is evident that the alteration in channel volume is influenced not only by the temperature but also by the flow rate of the experiment. Comparative analysis of experiments conducted under different flow conditions reveals a nonuniform dissolution pattern along the length of the channel, with increased reaction occurring at the inlet of the channel in instances of lower flow rates. These observations are consistent with previous simulation and experimental findings,^14,16^ which have shown that under reduced advection conditions, the longer time scale of advection becomes comparable to the time scale of transverse diffusion. This leads to species homogenization across the channel aperture and enhanced dissolution at the inlet. In contrast, at higher flow rates, transverse diffusion becomes less significant in species transport, resulting in a more uniform dissolution of minerals along the length of the channel.

Another noteworthy observation concerning different flow rates is that experiments conducted at lower flow rates while maintaining the same IFV exhibit a greater volume change, as depicted in Figure S4. This can be attributed to the short residence time of the reactive solution within the sample, limiting the rate of reactant transport to the mineral surface.^47^ Previous experimental findings^21,24,48^ indicate that the solution at the outlet of experiments, similar to the experimental conditions in this study, was highly undersaturated with respect to various reactive minerals. Consequently, reducing the flow rate increased the residence time and promoted species homogenization across the channel width. It consequently, enhanced the extent of reaction within the channel and reduced the IFV required to achieve a specific change in channel volume.^14^

The implications of these findings in the context of carbon storage are 2-fold. Utilizing low flow rates for the CO_2_ injection increases the likelihood of localized changes in the fracture network morphology. Excessive widening of fractures can adversely affect fracture network conductance, potentially resulting in fracture wall collapse under overburden stress.^21^ Conversely, employing higher flow rates not only facilitates increased CO_2_ injection into the reservoir but also ensures a more uniform alteration across a broader region of the fracture network. These observations are well aligned with the reported observations about the impact of flow rate (compared to the reaction rates) on the formation of wormholes during reactive transport. It has been reported that by change of flow rate from low to high flow rates, the dissolution patterns change from compact dissolution to wormholing.^49−52^

Analysis of individual 2D slices of sample “A” reveals that channel enlargement occurs uniformly across all experiments. This is attributed to the homogeneous nature of this sample, as illustrated in Figure 1a–c. The quartz and muscovite minerals are embedded within the calcite-based medium. The dissolution of the surrounding calcite during the experiment caused it to detach from the channel walls and exit the channel. As the initial channel aperture was larger than the size of these mineral particles, no closure or clogging of the channel was observed throughout the experiment due to this decohesion and migration. Conversely, the structural and mineralogical heterogeneity of sample “B” impacted channel enlargement differently. Figure 1c demonstrates the presence of an interconnected network of ankerite minerals that prevents their detachment after the dissolution of surrounding calcite minerals. This resulted in the development of a heterogeneous dissolution-altered structure around the initial channel, as depicted in Figure 2e,f.

Previous studies have reported the preferential dissolution of calcite compared with dolomite and silicate minerals in rocks with high clay contents. Noiriel et al.^21^ investigated argillaceous limestone containing 8% dolomite and over 25% clay minerals. They observed the formation of a dissolution-altered clay coating on the fracture walls, acting as a diffusive barrier to flow and mass transfer between calcite grains and the bulk solution, thereby significantly reducing the dissolution rate of the fracture walls. Ellis et al.^27^ studied the impact of CO_2_-saturated brine on cap rock integrity. Their sample consisted of 10% silicate minerals, including clay, and 90% carbonates (calcite and dolomite in approximately equal proportions). They also observed the formation of a silicate mineral-rich coating along the fracture wall due to the preferential dissolution of calcite in areas containing clay minerals. However, in the rest of the investigation domain, they observed only uneven erosion and increased fracture roughness due to the preferential dissolution of calcite over dolomite. Noiriel et al.^24^ studied another argillaceous limestone with only about 10% clay minerals. In these experiments, they observed increased roughness of the fracture walls without the formation of a dissolution-altered clay coating. Similarly, Gouze et al.^22^ reported a higher specific surface area and rougher fracture surface resulting from the dissolution of calcite compared to dolomite and clay minerals, without the development of a dissolution-altered clay coating.

It is evident that mineralogy, in particular the presence of clay minerals, plays a crucial role in the formation of the dissolution-altered layer. The mineralogical composition of the sample used in this study significantly differs from those of previous works. Sample “B” exclusively comprises carbonate minerals, which are prominent in reservoir rocks suitable for CO_2_ storage projects. Moreover, this study, to the best of our knowledge, is the first to characterize the evolution of the dissolution-altered layer (in the absence of clay minerals) in a 4D manner, thereby contributing significantly to the understanding of fluid flow evolution in fractured reservoirs during carbon storage.

### Characterization of Dissolution-Altered Layer

The altered area around the channel is shown in Figures S2 and 4a. The width or thickness of the dissolution-altered layer was determined by subtracting the radius of the initial channel in each cropped 2D slice (highlighted in yellow in Figure S2) from the radius of the cropped section.

**Figure 3:**
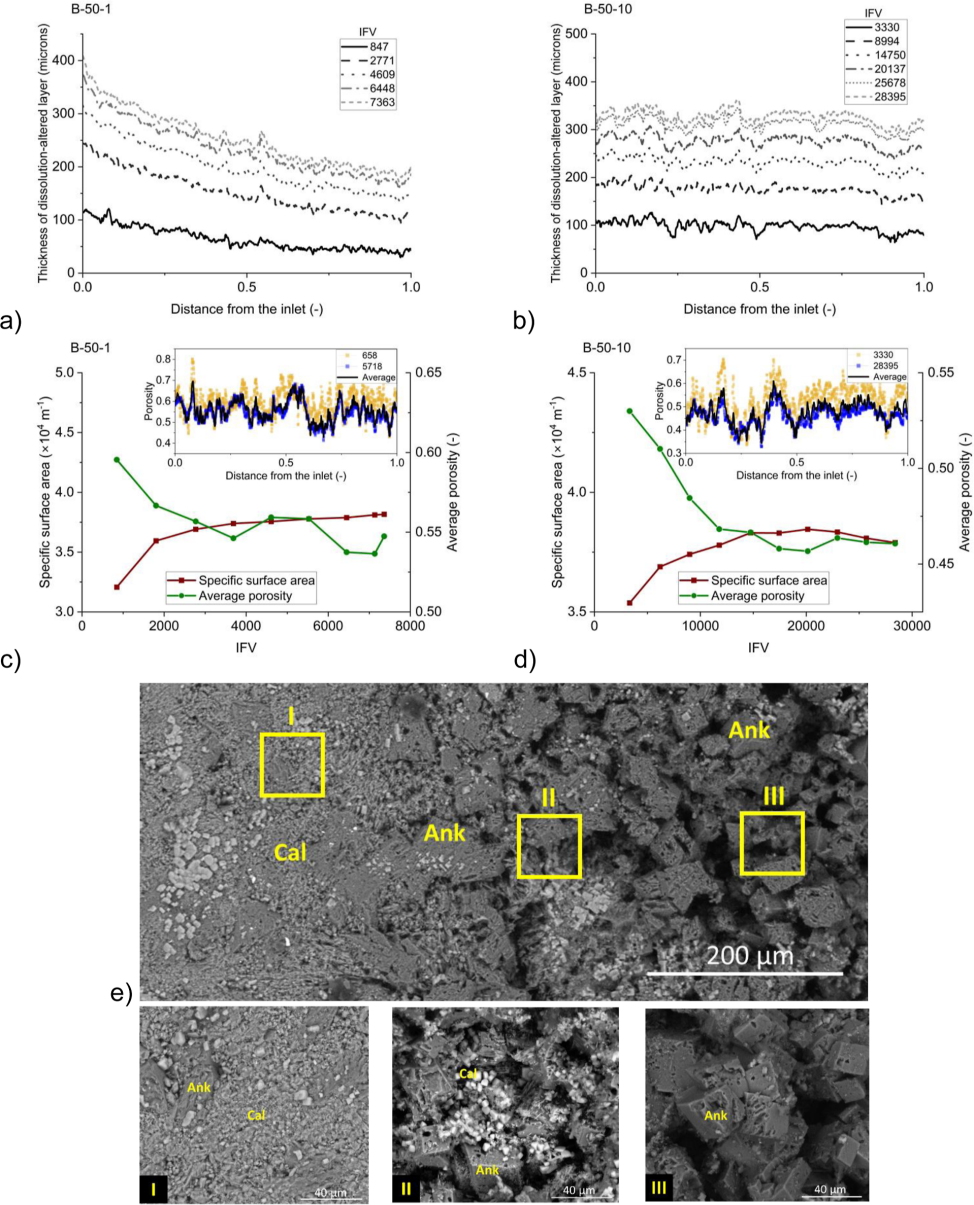
Thickness of the dissolution-altered layer at different times for experiments (a) B-50-1 and (b) B-50-10 are shown. Evolution of average porosity and specific surface area in the dissolution-altered layer for experiments (c) B-50-1 and (d) B-50-10 is presented. Insets depict the porosity profile along the channel length for the first scan (yellow), the last scan (blue), and the average porosity of all scans (black). (e) BSE images of a cross-section around the channel in sample B-50-1, depicts change of minerals from the intact area (I), the boundary (II), and the region that was in contact with CW, the dissolution-altered layer (III). Cal: calcite; Ank: ankerite.

Figure 3a,b present the temporal evolution of the dissolution-altered layer’s thickness profiles in the ankerite-containing samples. The thickness of the dissolution-altered layer increased over time in both cases. Under higher flow rates, the width demonstrated a nearly uniform enlargement along the flow direction. Conversely, as discussed in the preceding section, lower flow rates exerted a nonuniform influence, predominantly affecting the regions proximal to the inlet, resulting in an uneven expansion of the channel width and the associated dissolution-altered layer.

**Figure 4:**
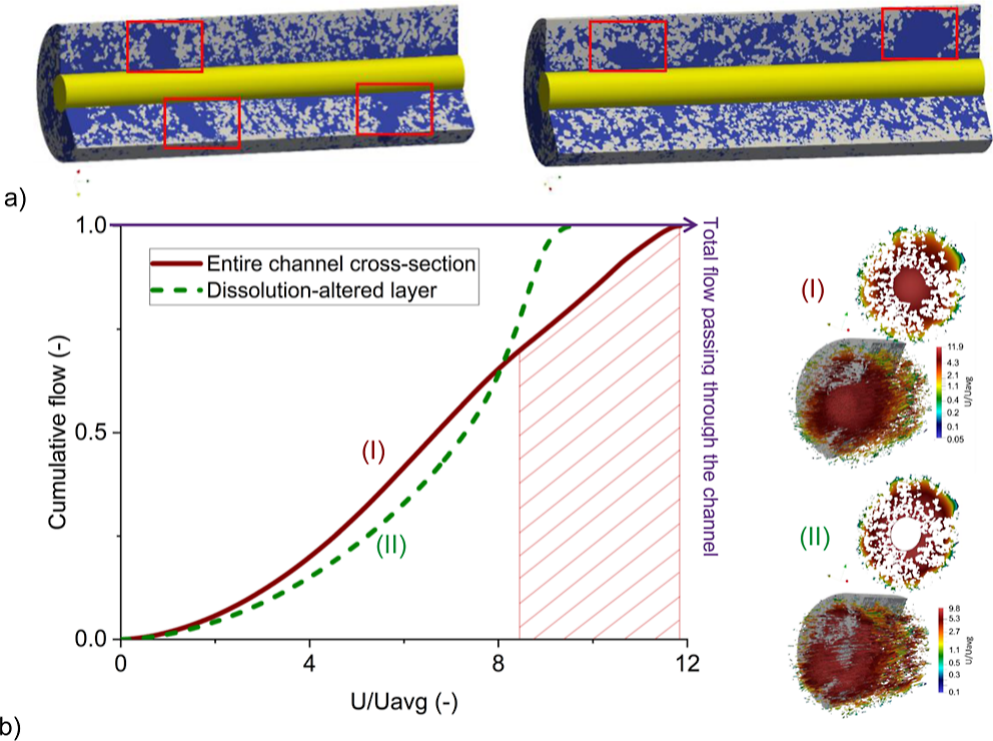
(a) Three-dimensional segmented representations of the dissolution-altered layer along the channel axis obtained from the B-50-10 experiment, revealing a heterogeneous distribution of solid and pore space. The creation of secondary peaks is highlighted by red rectangles. The initial channel is depicted in yellow, while the blue color represents the evolved channel area after the experiment and gray represents the remaining rock minerals. (b) The cumulative flow of the normalized velocity in the flow simulations (I) across the initial channel and the dissolution-altered layer (entire channel cross-section) and (II) only the dissolution-altered layer is presented. The hatched area represents the contribution of the initial channel to the cumulative flow. The normalized velocity distribution is depicted by using a logarithmic scale color bar.

Figure 3c,d provides insights into the dynamics of average porosity and average specific surface area within the dissolution-altered layer. In both flow rates, the porosity reduced eventually stabilizing at an approximately constant value. The insets showcase the porosity profiles in the dissolution-altered layer during the initial and final scans, alongside the average porosity across all scans. The porosity profiles demonstrated negligible dependence on flow rates, with changes along the channel axis primarily influenced by the initial distribution of ankerite minerals surrounding the channel. Intriguingly, the dissolution-altered layer displayed notable spikes of high porosity, which arise from the preferential dissolution of calcite over ankerite. This phenomenon led to the formation of vugs, as visually indicated by the red rectangles in Figure 4a. The orientation and expansion of these vugs depend on the spatial distribution of minerals.^21^

Moreover, the average specific surface area initially increased and then reached a near-constant value. The specific surface area enhancement can be attributed to increased roughness on the fracture wall^21,22,24,25^ and, in our case, the formation of the dissolution-altered layer. However, it is important to consider that the increase in the specific surface area does not directly translate into higher reactive surface area and higher dissolution rates. This observation is attributed to the presence of ankerite, which is significantly less reactive to calcite, while the specific surface area of calcite remains relatively constant.^22,27^ Backscattered electron (BSE) imaging was conducted on a cross-section of the channel and its surrounding area within sample B-50-1. These images illustrate a distinct inclination toward the dissolution of calcite minerals rather than ankerite within the dissolution-altered layer. This preference is evident when comparing images I to III in Figure 3e.

Previous studies^21,27^ assumed low porosity and permeability for the clay dissolution-altered layer based on calculated reaction rates. In contrast, in this study, we conducted numerical investigations to assess the fluid conductance of the ankerite dissolution-altered layer. To obtain an accurate 3D microstructure of the dissolution-altered layer, we conducted a complementary CT scan on sample B-50-10 after the experiment. The gray value cross sections, along with segmented 2D and 3D presentations of the dissolution-altered layer, are depicted in Figures S2 and 4a, respectively.

To calculate the permeability of the resulting microstructure, fluid flow simulations using the MRT-LBM method were performed. Two simulations were conducted with similar pressure gradients: the first simulation (4.b.I) involved flow occurring throughout the entire cross-section of the channel, including the initial channel and the dissolution-altered layer, while the second case (4.b.II) focused solely on 4.a (excluding the initial channel) to determine its permeability. The results revealed a permeability of 1.1 × 10^–10^ m^2^ for the first case and 4.3 × 10^–11^ m^2^ for the second simulation. These findings clearly demonstrate that the ankerite dissolution-altered layer in this study differs significantly from previous clay dissolution-altered layers in terms of flow restriction and diffusion dominance.

To further elucidate this distinction, Figure 4b presents the cumulative flow vs normalized velocity (velocity of each computational grid divided by the average velocity of the domain) for both simulation cases. Additionally, 3D velocity streamlines are depicted in a portion of the simulation domain, along with a 2D cross-section illustrating the velocity distribution. The cumulative flow represents the proportional contribution of each normalized velocity to the total flow rate. It is computed by summing the flow occurring at each specific velocity and dividing it by the average velocity of the simulated domain. The hatched area represents velocity values occurring in the initial channel area. It is evident that the central part of the channel contributed only partially to the total flow, while the dissolution-altered layer made a substantial proportion to the total flow rate in the first simulation case. In other words, species transport in the ankerite dissolution-altered layer was not only limited to diffusion. Due to the high fluid velocity in the dissolution-altered layer, advection played a significant role in transporting reactants from the mineral surfaces to the central part of the channel, or downstream further toward the outlet.

### Channel Permeability

Due to the very small pressure drop observed during the experiments, direct pressure measurements were impractical. Therefore, two methods were employed to estimate the permeability in this study. In the first method, permeability was calculated using the Hagen–Poiseuille equation, referred to as the analytical permeability . Using image analysis, the equivalent circle of the void space in each cross-section was calculated. By harmonic averaging of the diameter values along the channel,  values were calculated. Additionally, the computational permeability (*K*_c_) was calculated using the MRT-LBM. Results show an increase of permeability for all channels.

Figure S5 presents the calculated *K*_a_ and simulated *K*_c_ for experiments carried out using sample “A”. The results demonstrate that *K*_a_ consistently overestimated the permeability of the channel. This discrepancy can be attributed to the nonuniform diameter of the channel along its length, the influence of channel wall roughness, and averaging method along the channel. Nevertheless, analytical permeability estimation remains a viable and resource-efficient method when the channel profile changes uniformly along its length. In cases where nonuniform evolution occurs, the deviation between the two methods becomes significantly pronounced.

In the case of sample “B”, a thorough investigation was conducted by performing a scan with a longer duration and a high resolution of 3.3 μm to fully characterize the intricate 3D structure of the dissolution-altered layer. Subsequently, fluid flow within this microstructure was simulated, resulting in *K*_c_ = 1.1 × 10^–10^ m^2^ which indicates a significant error of over 200% compared to the analytical permeability, *K*_a_ = 3.2 × 10^–10^ m^2^. Clearly the analytical calculation of permeability has shortcomings for the case of sample “B” since the effect of the dissolution-altered layer cannot be captured.

### Upscaled Dissolution Rate

**Figure 5:**
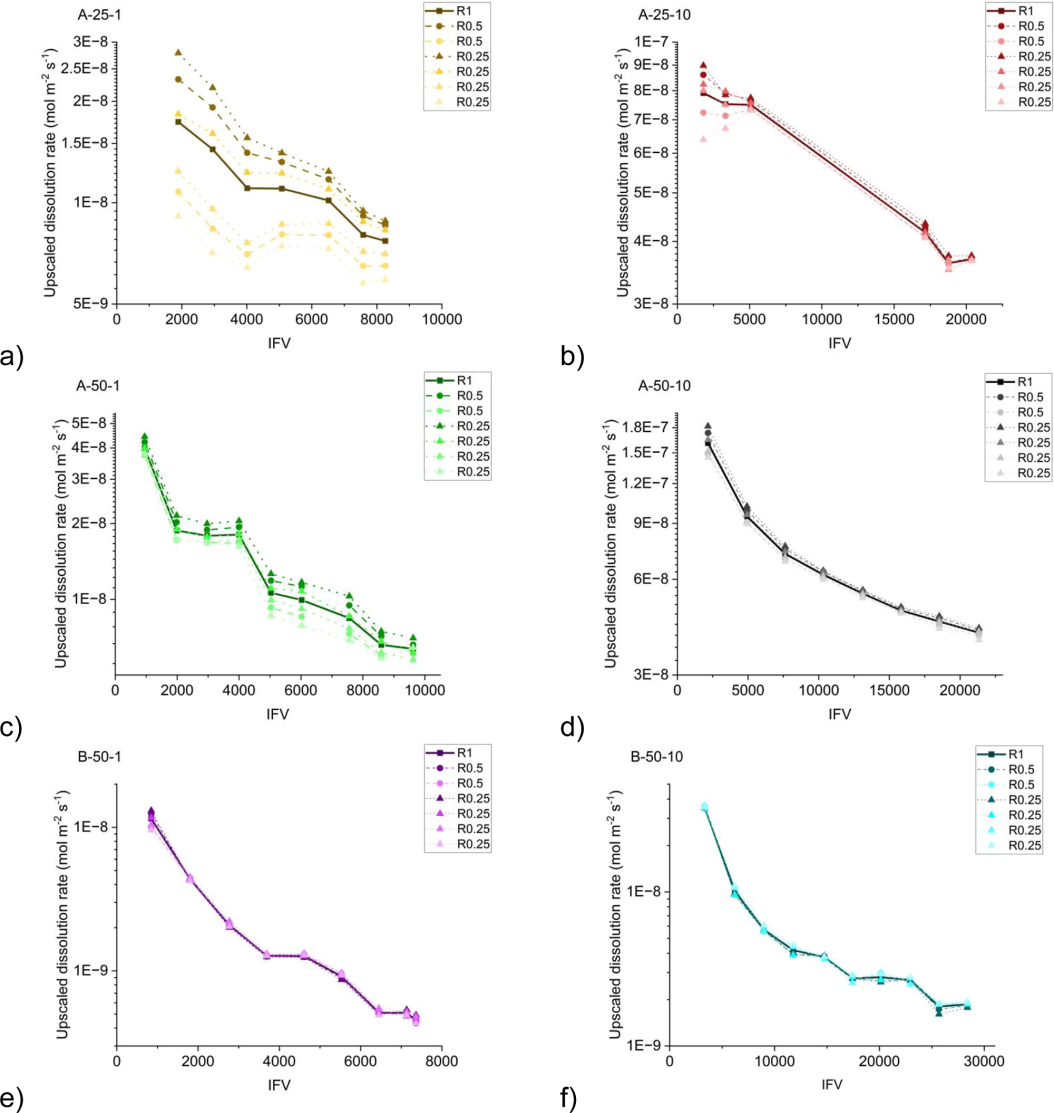
Upscaled dissolution rate is presented for experiments: (a) A-25-1, (b) A-25-10, (c) A-50-1, (d) A-50-10, (e) B-50-1, and (f) B-50-10. *R*_1_, *R*_0.5_, and *R*_0.25_ are shown with square, circle, and triangle symbols, respectively. Moreover, the darker colors represent a location closer to the inlet. The bulk dissolution rates for samples “A” at 25 and 50 °C under the experimental pressure were calculated as 4.8 × 10^–4^ and 7.1 × 10^–4^ mol m^–2^ s^–1^, respectively. The bulk dissolution rate for sample “B” was calculated as 6.1 × 10^–4^ mol m^–2^ s^–1^ at 50 °C.

As previously discussed, the upscaled dissolution rate was calculated at various locations within the channel, spanning different length scales. The results for samples “A” and “B” are presented in Figure 5. The calculated upscaled dissolution rates in this study exhibited a significant deviation, up to 4 orders of magnitude, from the intrinsic reaction rates measured in batch experiments. This disparity can be attributed to the mass transfer limitation between the fast-flowing channel and the surrounding matrix, as well as the limited surface area available for interaction with the reactive fluid.^23,44,47,53^

Across all cases, a consistent trend is observed, whereby the upscaled dissolution rate decreased as the experiment progressed. Table S2 in the Supporting Information demonstrates that increasing the channel diameter resulted in a lower fluid velocity within the channel and a reduction in *Pe* up to 1 order of magnitude. This reduction in fluid velocity negatively impacted species transport to and from reactive sites. Additionally, the diffusion time scale  increased as the channel diameter expanded, resulting in slower homogenization of species across the channel width and consequently a reduced reaction rate. With a similar explanation, in experiments with a higher flow rate, increasing the fluid velocity in the channel increased the upscaled dissolution rate. Higher fluid velocities promoted increased mass transfer and better mixing of the reacting solution around the calcite surface. This increased mass transfer helped maintain a fresh supply of reactive species at the surface, ensuring a higher dissolution rate.

Furthermore, when comparing the results of sample “A” with respect to temperature, it was observed that, at both flow rates, the upscaled dissolution rate is smaller for the lower temperature. This observation aligns with the expected behavior, as the intrinsic dissolution rate of calcite is known to be lower at 25 °C compared to 50 °C.^46^

As discussed in the “characterization of dissolution-altered layer” section, experimental findings suggested that the increased surface area in the dissolution-altered layer is predominantly associated with ankerite with 1 order of magnitude lower reactivity compared to calcite. This raises the question of whether this excess surface area should be accounted for in the calculation or not. The answer to this question largely depends on how the development of the dissolution-altered layer is integrated into Darcy-scale models and how these increased values are incorporated. Figure 5e,f present the upscaled dissolution rates for sample “B”, where the excess surface area resulting from the development of the dissolution-altered layer was considered in the calculation of the upscaled dissolution rate. Figure S5 only incorporated the average surface area expansion calculated using the average diameters obtained from image analysis (Table S2 in the Supporting Information). An intriguing observation can be made from these figures, including the surface area contributed by the dissolution-altered layer arbitrarily decreased the upscaled dissolution rate. These results emphasized the importance of adopting a proper approach for this implementation, as the upscaled dissolution rate can vary by up to 1 order of magnitude depending on the chosen approach.

By using a similar method to calculate the average surface area, a comparison can be made between the upscaled dissolution rates of samples “A” and “B”. Comparing the results in Figure 5c,d with S6 revealed only a slight difference in the values of the upscaled dissolution rates. Previous studies on rock types with significant clay content^21,27^ have indicated that the development of the clay dissolution-altered layer is the main factor causing retardation in the dissolution rate. In contrast, we did not observe a similar trend. The high porosity and permeability of the ankerite dissolution-altered layer in our case may explain this behavior as advection could still play a crucial role in transporting species to and from the reactive surfaces.

Upon further examination of the results, it became evident that the upscaled dissolution values were influenced by both the spatial location and length scale of averaging. In cases where the channel profile exhibited uniform changes along its length, the impact of these factors was minimal and a single upscaled dissolution value could adequately capture the overall rate of change within the channel. However, at lower flow rates when the channel profile developed nonuniformly along the length of the channel, the significance of the length scale and spatial location became more pronounced. Under such conditions, where a greater proportion of the dissolution occurs in proximity to the inlet, a clear trend emerged *R*_0.25_ > *R*_0.5_ > *R*_1_. This observation indicates that the upscaled dissolution values gradually increased as the length scale of the averaging decreased. The magnitude of this difference can be substantial, with variations of up to 2-fold observed, as exemplified in the case of A-25-1.

## Environmental Implications

Developing robust predictive and monitoring techniques remains crucial in ongoing research concerning CO_2_ storage. The observed discrepancy between upscaled dissolution rates from experimental setups and those in batch experiments, alongside the scale-dependency of dissolution rates, highlights a critical challenge in accurately predicting CO_2_-induced dissolution rates of CO_2_ within geological formations. This discrepancy, rooted in the limitations of available reactive surface area in high-velocity fractures, emphasizes the shortcomings in available models to predict the dissolution. Clearly the experiments show that within reservoir conditions after the dissolution of CO_2_ in brine, dissolution of carbonate rocks takes place in short experimental time scales. This indicates the potential risk for the long-term injection of CO_2_ which may impost safety issues regarding the integrity of the reservoir specially near the well bore during the long-term CO_2_ injection.

The observations of nonuniform fracture dissolution at varying flow rates and the influence of rock mineralogy on the upscaling process carry significant implications. At higher flow rates (Péclet number ≈2 × 10^4^ and *Da* number ≈10^–3^), the uniform dissolution pattern suggests the potential viability of estimating permeability changes from channel volume alterations. Conversely, at lower flow rates (Péclet number ≈2 × 10^3^ and *Da* number ≈10^–2^), the nonuniform dissolution of fractures signifies the interplay between fluid dynamics and reactive transport within fractured carbonate reservoirs.

The challenges posed by rock mineralogy, particularly the presence of ankerite, in the scaling up process add a layer of complexity to the CO_2_ storage modeling. Dissolution-altered layers in ankerite-bearing carbonates, as revealed in the study, diverge from clay-induced alterations. While clay alterations typically impede fluid movement due to reduced permeability and porosity, the ankerite-induced alterations demonstrate high permeability and porosity without significantly affecting reaction rates within the experimental time frame. This observation challenges previous assumptions about the impact of dissolution-altered layers on fluid conductance and reactive transport dynamics within fractured carbonate reservoirs. Results demonstrate that with increase of flow rate, the discrepancy between the reaction rates resulted from different scales decreases. This is aligned with the dissolution pattern that indicates with increase of flow rate, at different scales more of less similar dissolution rates will be observed.

## Supporting Information Available

Further information on the experimental procedure; details of the experimental setup and rock samples; list of experiments; details of the image processing; additional results for upscaled dissolution rate in rock B; and comparison between the analytical and numerical calculations of the channel permeability (PDF)
