# Supplementary material for: Exploring Carbonate Rock Dissolution Dynamics and the Influence of Rock Mineralogy in CO2 Injection
Source: Environ Sci Technol. 2024 Jan 17;58(6):2728–38. doi: 10.1021/acs.est.3c06758 (PMC10867842; doi:10.1021/acs.est.3c06758)
Supplement: Supplementary file 1 — es3c06758_si_001.pdf [file es3c06758_si_001.pdf]

Supporting Information:
Exploring Carbonate Rock Dissolution Dynamics and the Influence of Rock Mineralogy during CO_2 Injection

Javad Shokri[1], Matthias Ruf[2], Dongwon Lee[2], Saleh Mohammadrezaei[1], Holger Steeb[2], and Vahid Niasar*[1]

[1]Department of Chemical Engineering, University of Manchester, Oxford Road, Manchester, M13 9PL, United Kingdom

[2]Institute of Applied Mechanics (MIB), Stuttgart University, Pfaffenwaldring 7, 70569 Stuttgart, Germany

Summary

The Supporting Information spans thirteen pages and presents two tables and six figures along the detailed information on the experimental setup, image processing and additional results.

*vahid.niasar@manchester.ac.uk

Contents

S1 Rock Samples **S4**

S2 Experimental Apparatus and Method **S4**

S2.1 Experimental setup S4

S2.2 3D Imaging S5

S2.3 Image post-processing S7

S3 Péclet and Damköhler Numbers, and Upscaled Dissolution Rates **S7**

S4 Additional Results and Discussion **S9**

S4.1 Evolution of the channel morphology S9

S4.2 Channel permeability S9

S4.3 Upscaled dissolution rate S9

List of Figures

S1 a) The diameter of the fracture is measured using 360 and 1440 projections before starting the experiment A-25-10. b) The porosity of the dissolution-altered layer is measured using scans performed with 1440 projections (6.5 µm voxel size) at the end of the B-50-10 experiment, and 2400 projections (5.3 µm voxel size) using Versa 620 imaging system. c) complementary to the porosity profile along the axis of the flow, cross sections from different locations are shown for visual comparison of the micro-structure inside the dissolution-altered layer S6

S2 2D slices of the dissolution-altered layer in grayscale (top row), pore space evolved during experiment (middle row), and the solid phase in the bottom row. The yellow color shows the initial fracture. S8

S3 Demonstration of upscaling of dissolution rate at different locations and length scales. S8

S4 The cumulative channel volume change and the relative volume change for all experiments are shown. S9

S5 Calculated analytical permeability (K_a) and computed permeability (K_c) are presented for experiments conducted on sample "A". The values displayed on each data point represent the difference between K_a and K_c. S10

S6 The upscaled dissolution rate is illustrated for experiments conducted on sample "B" without including the surface area of the dissolution-altered layer: a) B-50-1, b) B-50-10. The bulk dissolution rate for sample "B" was calculated as 6.1×10^{-4} mol m^{-2} s^{-1}. S10

List of Tables

S1 Number of projections in experimental cases S5
S2 The evolution of the equivalent diameter, Péclet and Damköhler numbers for experiments are presented. S11

S1 Rock Samples

Two distinct rock types sourced from carbonate reservoirs were used in this study. From each rock sample, cylindrical core plugs with a diameter of 6.4 mm were cut. These plugs were then cut and polished to attain a length of approximately 8.5 mm.

Fractures, typically induced through tensile fracturing or saw cutting methodologies, present challenges owing to their inherent variability and limited controllability. The first method's inherent randomness in initiating fractures and the lack of strict control over fracture aperture in both approaches pose significant limitations. Moreover, the influence of confining pressure further complicates the scenario, potentially altering or even entirely closing the fracture during experimentation. Addressing these complexities and aiming to maintain post-experiment access for additional scans (as detailed in section S2.2 regarding scans with Xradia Versa 620), a decision was made to create controlled-aperture holes at the center of carbonate core plugs to mimic fracture. This ensured consistent fracture properties across the core plugs, improving reproducibility while isolating the impact of confining pressure on fracture size. This strategy aimed to ensure consistent observations and measurements throughout the experimentation process and afterward.

To create these fractures, a drill bit with a nominal diameter of 300 µm and a length of 5 mm was employed. The process was carried out using a computer numerical control (CNC) milling machine, operating at a speed of 10000 rounds per minute. Following the drilling, Soxhlet extraction was used to remove contaminants. Subsequently, the core plugs underwent immersion in a 1:1 mixture of ethanol and isopropyl alcohol for several days to eliminate any residual drilling fluid or potential contaminants. Finally, the plugs were dried under controlled conditions at a temperature of 70°C.

S2 Experimental Apparatus and Method

S2.1 Experimental setup

The flow cell was made of polyether ether ketone (PEEK) which is X-ray transparent. The design is similar to the one presented in [1], but was adapted for core plugs with a diameter of 6.4 mm. The flow cell was enclosed in a heating chamber made of polytetrafluoroethylene (PTFE) to minimize X-ray absorption of the heating system. A heating circulator regulated the experiment's temperature by flowing the heating liquid into the heating chamber around the flow cell and injection pump. Two ISCO pumps and one CETONI syringe pump were used to inject and receive carbonated water (CW) and apply the confining pressure, respectively.

All experiments were performed using a brine solution representative of a typical saline aquifer containing 1 M NaCl. To achieve equilibrium, brine and an excess amount of CO_2 (50% more than the estimated amount based on experimental data [2]) were transferred and kept at the experimental condition (10 MPa, 25°C or 50°C) in the injection pump. The excess amount of CO_2 was then removed from the injection pump under constant pressure conditions before the experiment. The core plug was wrapped in aluminum foil to

Table S1: Number of projections in experimental cases

ID	Number of Projections
A-25-1	360
A-25-10	360
A-50-1	1440
A-50-10	720
B-50-1	1440
B-50-10	1440

prevent CO_2 crossover to the confining area. It was then placed in a silicone sleeve and secured using a heat-shrinkable tube before mounting it into the flow cell on the rotation stage. The sample was saturated with still brine (with the same composition as CW), and the pressure was increased to 10 MPa. The flow cell and injection pump were heated to the desired temperature, and the setup was kept at these conditions for several hours to ensure complete saturation and temperature equilibrium. The first X-ray scan of the core plug was performed after ensuring full saturation and was used as the base scan (without any reaction) for each sample. Experiments were done at two flow rates of 1 µL min^{-1} and 10 µL min^{-1}.

S2.2 3D Imaging

In order to observe the dissolution over time, continuous time-lapse imaging was conducted using µXRCT [3, 4]. For this purpose, a modular laboratory-based µXRCT system was designed as described in detail in [5]. The imaging setup was configured to capture the complete fracture in the axial direction of the sample and the entire width of the sample, resulting in an isotropic voxel size of 6.5 µm, employing a Dexela 1512NDT detector.

The X-ray tube was operated at a voltage of 100 kV and a current of 100 µA. To enhance the image quality, two slightly shifted projection images were acquired and subsequently averaged. The number of equidistant projection angles employed for a full rotation of the sample varied based on the speed of the chemical reaction and the microstructure of the sample. Specifically, values of 360, 770, and 1440 projection angles were utilized (Table S1), corresponding to scan periods of 22, 41, and 75 minutes, respectively. It is worth noting that the utilization of a lower number of projection angles for certain scans was justified by the fact that only the central area of the fracture was of interest specially in experiments performed with sample "A" where no dissolution-altered layer was created. Thus, the quantification of fracture evolution remained unaffected. Figure S1.a visually illustrates that even with fewer projections, adequate capture of the fracture diameter was achieved when compared to scans utilizing a higher number of projections.

The reconstruction of all acquired 3D images was performed using the Feldkamp-Davis-Kress (FDK) reconstruction algorithm [6], implemented in the Octopus Reconstruction software (Version 8.9.4-64 bit) [7]. During the reconstruction process, typical artifacts such as ring artifacts and beam hardening effects were compensated for, ensuring an accurate representation of the fracture evolution within the reconstructed images.

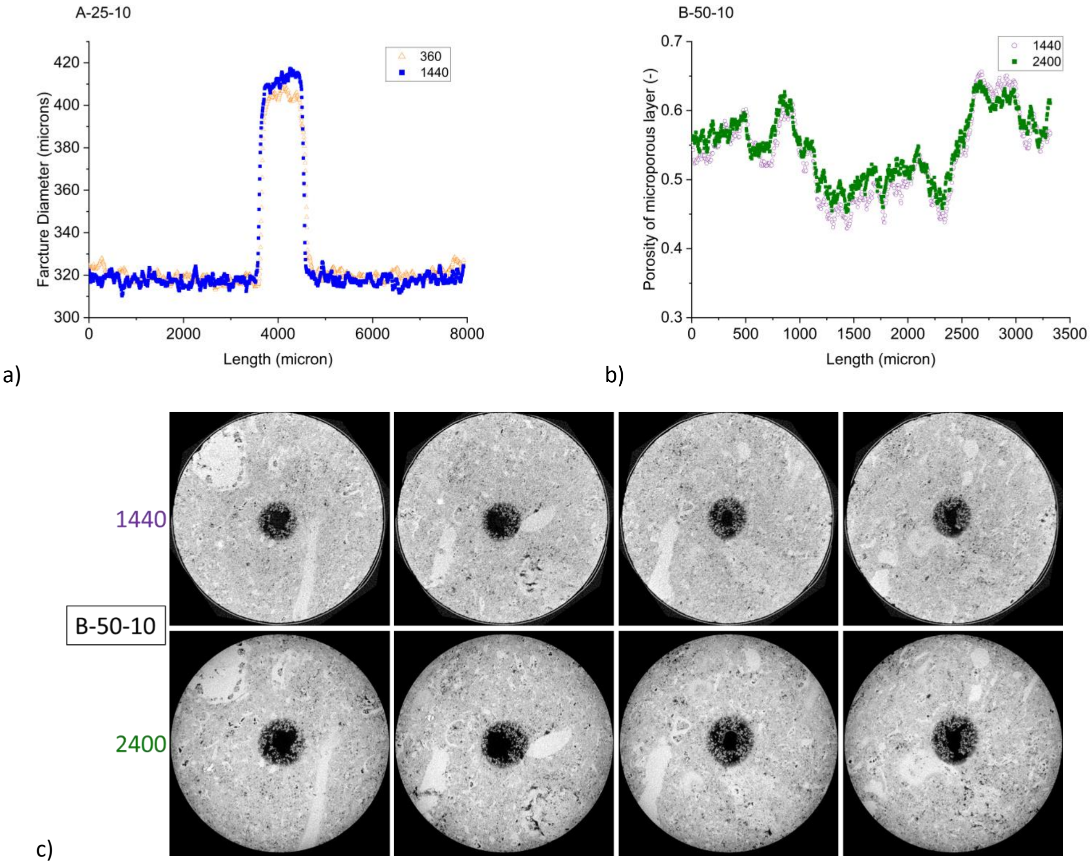

Figure S1: a) The diameter of the fracture is measured using 360 and 1440 projections before starting the experiment A-25-10. b) The porosity of the dissolution-altered layer is measured using scans performed with 1440 projections (6.5 µm voxel size) at the end of the B-50-10 experiment, and 2400 projections (5.3 µm voxel size) using Versa 620 imaging system. c) complementary to the porosity profile along the axis of the flow, cross sections from different locations are shown for visual comparison of the micro-structure inside the dissolution-altered layer .

Moreover, complementary CT scans on sample B-50-10 after completing the experiment was performed. Xradia Versa 620 imaging system, was employed to achieve a 5.3 and 3.3-micron isometric voxel size. A total of 2400 projections, with an exposure time of 2 seconds and a total scan time of about 4 hours, were acquired to fully capture the dissolution-altered structure. The assessment of time-lapse imaging's efficacy in capturing the microstructure of the dissolution-altered layer was also assessed. Figure S1.b-c presents a comparative analysis, showcasing that the porosity of the dissolution-altered layer remained nearly identical between scans conducted with 1440 projections using continuous time-lapse imaging setup and scans performed with 2400 projections utilizing the Xradia Versa 620 imaging system. This visual representation underscores the similarity in porosity measurements obtained through differing projection quantities, indicating a consistent portrayal of the dissolution-altered layer's structural characteristics despite variations in imaging techniques and projection counts.

S2.3 Image post-processing

In order to cope with the inherent noise of µXRCT reconstructed images, the total variation filtering method [8, 9] was employed on the images as a means of an edge-preserving denoising technique. Subsequently, the Otsu method [10], which is one of the automated threshold segmentation techniques, was applied to the filtered images. This allowed the filtered images to be segmented into solid and void phases. The isolated and misclassified voxels, resulting from remaining artifacts, were further reassigned by checking their connectivity from top to bottom, assuming that no voxels can float. Based on the segmented 3D images, a quantification of the fracture evolution over position and time was carried out.

To determine the fracture evolution over time, the region of interest was restricted to the fracture and its close proximity in each 2D slice of CT images. Image analysis was utilized to segment images into solid and fracture spaces. The volume of the fracture was calculated by counting the number of pixels in the fracture region multiplied by the thickness of each slice. By subtracting the fracture volume in each slice from the same slice in the base scan (i.e. saturated with brine at the beginning of the experiment), the change in fracture volume was calculated.

Moreover, the dissolution-altered layer was segmented into solid and pore spaces. Porosity was quantified by dividing the number of pixels belonging to the pore space by the total number of pixels in the cropped 2D cross-section. Furthermore, the specific surface area in each 2D slice was calculated by dividing the total number of pixels on the pore-solid boundary by the total volume of the cropped image. The quality of the image segmentation technique is shown in Figure S2 for various 2D slices along the fracture length.

S3 Péclet and Damköhler Numbers, and Upscaled Dissolution Rates

The Péclet number (Pe) compares the rate of advection to the rate of diffusion, while the Damköhler number (Da) represents the ratio between the timescales of advection and reaction [11, 12, 13]. Quartz and muscovite were excluded from kinetic rate calculations due to their significantly lower intrinsic reaction rates in contrast to calcite and ankerite [14, 15]. Calcite's intrinsic reaction rates at 25°C and 50°C under experimental pressure were considered as 5.6×10^{-4} and 8.1×10^{-4} mol m^{-2} s^{-1}, respectively [16]. Moreover, ankerite's estimated intrinsic reaction rate was 5.1×10^{-5} mol m^{-2} s^{-1} based on findings from [17].

Figure S3 illustrates how various upscaled dissolution rates have been calculated from different locations and different lengths. This will result in one upscaled dissolution rate for the entire length of the fracture (R_1), two rate values for the half-length of the fracture ($R_{0.5}$), and four rate values for each quarter of the fracture ($R_{0.25}$), where, R [mol m^{-2} s^{-1}] denoted as the upscaled or effective dissolution rate. ρ_s represents the density of pure minerals, with values of 2711 kg m^{-3} for calcite and 3000 kg m^{-3} for ankerite [18], M_s is the molecular weight, with values of 0.1 kg mol^{-1} for calcite and 0.2 kg mol^{-1} for ankerite, In this study, muscovite and quartz are considered non-reactive compared to calcite and ankerite due to their significantly lower intrinsic reaction rates. Therefore, their contributions to the reactive volume change are excluded.

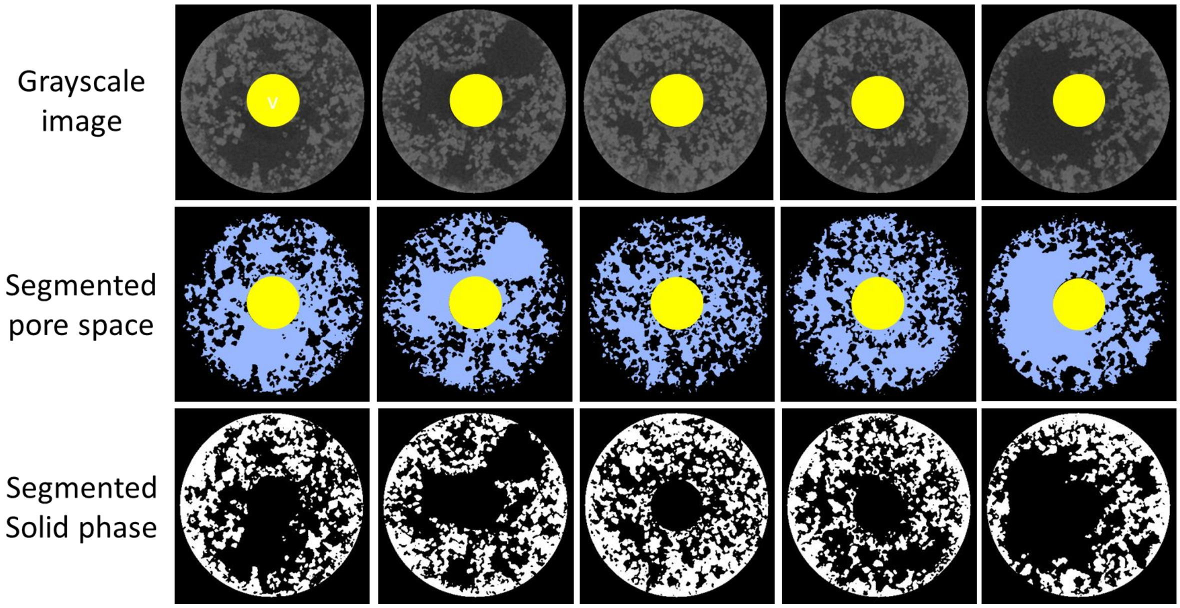

Figure S2: 2D slices of the dissolution-altered layer in grayscale (top row), pore space evolved during experiment (middle row), and the solid phase in the bottom row. The yellow color shows the initial fracture.

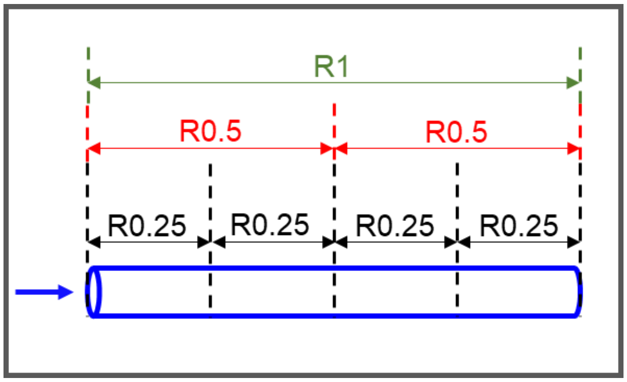

Figure S3: Demonstration of upscaling of dissolution rate at different locations and length scales.

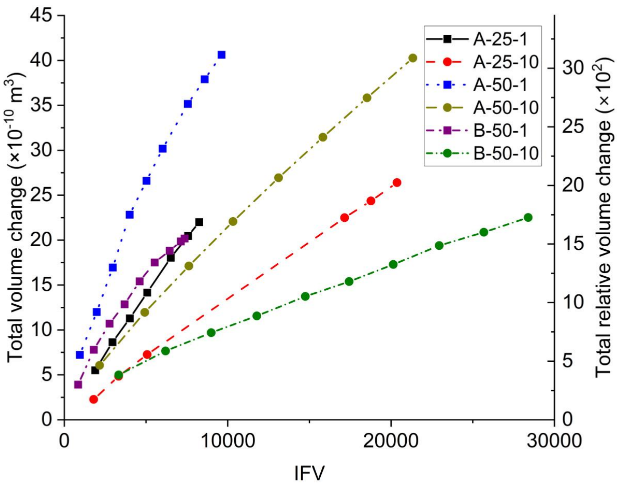

Figure S4: The cumulative channel volume change and the relative volume change for all experiments are shown.

S4 Additional Results and Discussion

S4.1 Evolution of the channel morphology

Figure S4 shows the total cumulative channel volume change during each experiment and the relative volume change of the channel (defined as the volume change divided by the initial channel volume).

S4.2 Channel permeability

Two methods were employed to estimate permeability due to impractical direct pressure measurements caused by minimal pressure drop in the experiments. The first method used the Hagen-Poiseuille equation (analytical permeability) and MRT-LBM to calculate permeability. Results showed increased permeability in all channels, but discrepancies arose between calculated analytical permeability (K_a) and computational permeability (K_c). Figure S5 shows the results for sample A and the rest of the discussion can be found in the manuscript in section "channel permeability".

S4.3 Upscaled dissolution rate

Figure S6 depicts the upscaled dissolution rate for experiments performed on sample "B" without including the surface area of the dissolution-altered layer.

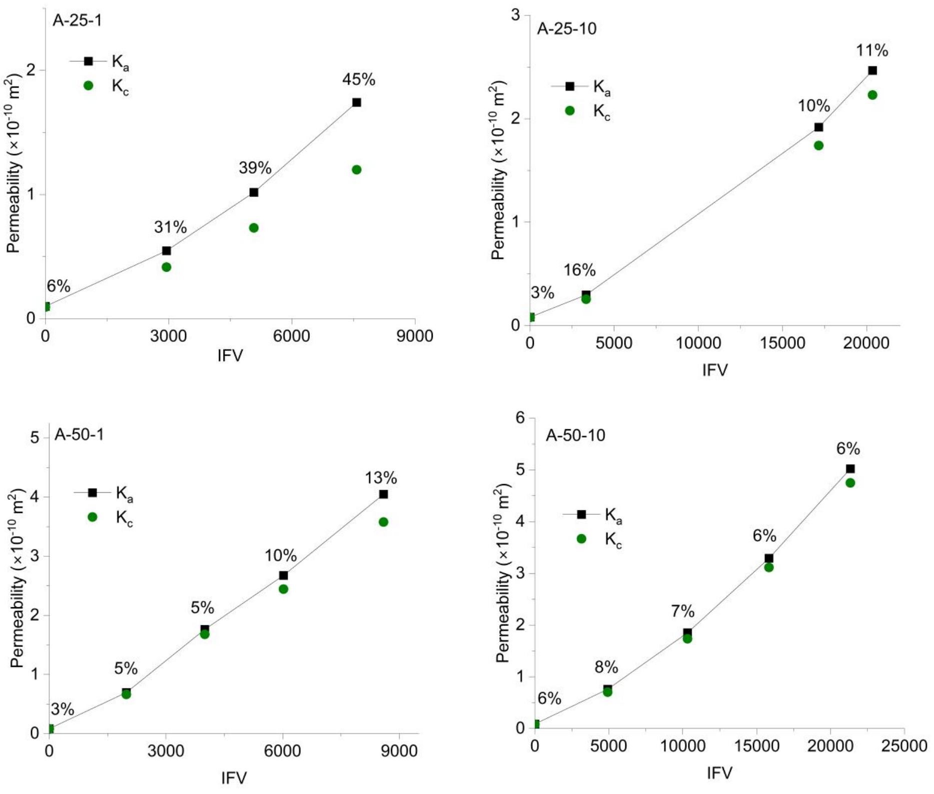

Figure S5: Calculated analytical permeability (K_a) and computed permeability (K_c) are presented for experiments conducted on sample "A". The values displayed on each data point represent the difference between K_a and K_c.

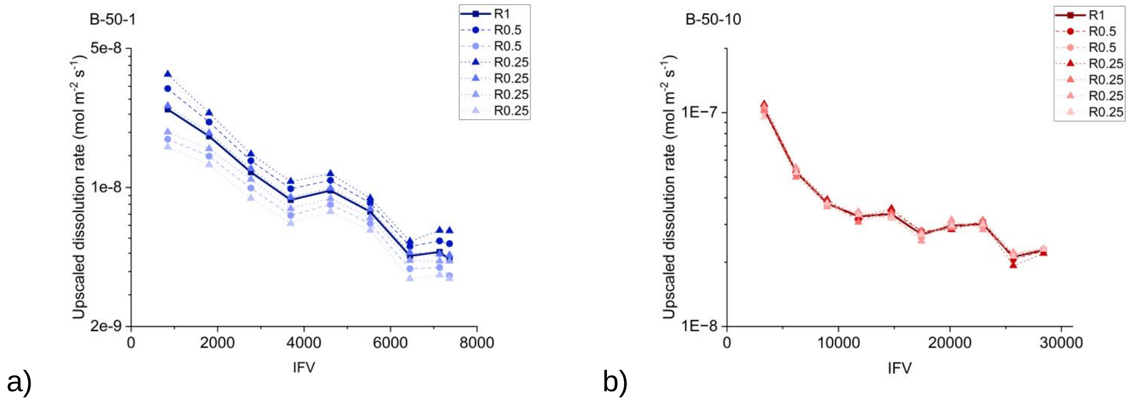

Figure S6: The upscaled dissolution rate is illustrated for experiments conducted on sample "B" without including the surface area of the dissolution-altered layer: a) B-50-1, b) B-50-10. The bulk dissolution rate for sample "B" was calculated as 6.1×10^{-4} mol m^{-2} s^{-1}.

Table S2: The evolution of the equivalent diameter, Péclet and Damköhler numbers for experiments are presented.

ID	IFV	$\mathcal{D}$ (micron)	Pe	Da
A-25-1	0	329	2216	7.1x10^{-2}
	1890	444	1185	1.3x10^{-1}
	4011	532	803	2.0x10^{-1}
	6517	624	581	2.7x10^{-1}
	8259	673	499	3.2x10^{-1}
A-25-10	0	313	24304	6.5x10^{-3}
	1801	379	16500	9.6x10^{-3}
	5073	475	10561	1.5x10^{-2}
	17159	689	5033	3.1x10^{-2}
	20362	734	4438	3.6x10^{-2}
A-50-1	0	314	2430	9.5x10^{-2}
	949	460	1130	2.0x10^{-1}
	2966	603	658	3.5x10^{-1}
	5031	716	465	5.0x10^{-1}
	7560	801	369	6.3x10^{-1}
	9622	852	326	7.1x10^{-1}
A-50-10	0	318	23633	9.8x10^{-3}
	2163	449	11864	1.9x10^{-2}
	7624	621	6215	3.7x10^{-2}
	10324	684	5122	4.5x10^{-2}
	15830	790	3842	6.0x10^{-2}
	21336	878	3111	7.4x10^{-2}
B-50-1	0	333	2155	1.0x10^{-1}
	847	412	1391	1.6x10^{-1}
	2771	521	864	2.6x10^{-1}
	4609	584	684	3.2x10^{-1}
	6448	626	594	3.7x10^{-1}
	7363	641	565	3.9x10^{-1}
B-50-10	0	318	23520	9.4x10^{-3}
	3330	433	12741	1.7x10^{-2}
	8994	518	8898	2.5x10^{-2}
	14750	582	7068	3.1x10^{-2}
	20137	632	5984	3.7x10^{-2}
	28395	700	4883	4.5x10^{-2}

References

[1] Florian Fusseis, Holger Steeb, Xianghui Xiao, Wen lu Zhu, Ian B. Butler, Stephen Elphick, and Urs Mäder. A low-cost X-ray-transparent experimental cell for synchrotron-based X-ray microtomography studies under geological reservoir conditions. *Journal of Synchrotron Radiation*, 21(1):251–253, dec 2014. doi: 10.1107/s1600577513026969.

[2] Zhenhao Duana; Rui Sun. An improved model calculating CO2 solubility in pure water and aqueous NaCl solutions from 273 to 533 K and from 0 to 2000 bar. *Chemical Geology*, 1(193):257–271, 2003. ISSN 17415144. doi: 10.1504/ijhtm.1999.001056.

[3] S.C. Garcea, Y. Wang, and P.J. Withers. X-ray computed tomography of polymer composites. *Composites Science and Technology*, 156:305–319, mar 2018. doi: 10.1016/j.compscitech.2017.10.023.

[4] Parmesh Gajjar, Jakob S. Jørgensen, Jose R. A. Godinho, Chris G. Johnson, Andrew Ramsey, and Philip J. Withers. New software protocols for enabling laboratory based temporal CT. *Review of Scientific Instruments*, 89(9):093702, sep 2018. doi: 10.1063/1.5044393.

[5] Matthias Ruf and Holger Steeb. An open, modular, and flexible micro x-ray computed tomography system for research. *Review of Scientific Instruments*, 91(11):113102, 2020.

[6] L. A. Feldkamp, L. C. Davis, and J. W. Kress. Practical cone-beam algorithm. *Journal of the Optical Society of America A*, 1(6):612, jun 1984. doi: 10.1364/josaa.1.000612.

[7] J. Vlassenbroeck, M. Dierick, B. Masschaele, V. Cnudde, L. Van Hoorebeke, and P. Jacobs. Software tools for quantification of X-ray microtomography at the UGCT. *Nuclear Instruments and Methods in Physics Research Section A: Accelerators, Spectrometers, Detectors and Associated Equipment*, 580(1): 442–445, sep 2007. doi: 10.1016/j.nima.2007.05.073.

[8] Leonid I. Rudin, Stanley Osher, and Emad Fatemi. Nonlinear total variation based noise removal algorithms. *Physica D: Nonlinear Phenomena*, 60(1-4):259–268, nov 1992. doi: 10.1016/0167-2789(92)90242-f.

[9] David Strong and Tony Chan. Edge-preserving and scale-dependent properties of total variation regularization. *Inverse Problems*, 19(6):S165–S187, nov 2003. doi: 10.1088/0266-5611/19/6/059.

[10] Nobuyuki Otsu. A threshold selection method from gray-level histograms. *IEEE Transactions on Systems, Man, and Cybernetics*, 9(1):62–66, jan 1979. doi: 10.1109/tsmc.1979.4310076.

[11] Christopher N Fredd and H Scott Fogler. Influence of transport and reaction on wormhole formation in porous media. *AIChE journal*, 44(9):1933–1949, 1998.

[12] Fabrice Golfier, Cesar Zarcone, Brigitte Bazin, Ronald Lenormand, Didier Lasseux, and Michel Quintard. On the ability of a darcy-scale model to capture wormhole formation during the dissolution of a porous medium. *Journal of fluid Mechanics*, 457:213–254, 2002.

[13] S Kim and J C Santamarina. Geometry-coupled reactive fluid transport at the fracture scale : application to CO 2 geologic storage. *Geofluids*, pages 329–341, 2016. doi: 10.1111/gfl.12152.

[14] Kristin Lammers, Megan M Smith, and Susan A Carroll. Muscovite dissolution kinetics as a function of ph at elevated temperature. *Chemical Geology*, 466:149–158, 2017.

[15] Patricia M Dove and Stephen F Elston. Dissolution kinetics of quartz in sodium chloride solutions: Analysis of existing data and a rate model for 25 c. *Geochimica et Cosmochimica Acta*, 56(12):4147–4156, 1992.

[16] Cheng Peng, John P Crawshaw, Geoffrey C Maitland, and JP Martin Trusler. Kinetics of calcite dissolution in co2-saturated water at temperatures between (323 and 373) k and pressures up to 13.8 mpa. *Chemical Geology*, 403:74–85, 2015.

[17] Cheng Peng, Benaiah U Anabaraonye, John P Crawshaw, Geoffrey C Maitland, and JP Martin Trusler. Kinetics of carbonate mineral dissolution in co 2-acidified brines at storage reservoir conditions. *Faraday discussions*, 192:545–560, 2016.

[18] David Barthelmy. Mineralogy database. *http://webmineral. com/*, 2007.
